# Prevalence and associated factors of supine sleep position in 3-month-old infants: findings from the 2015 Pelotas (Brazil) Birth Cohort

**DOI:** 10.1186/s12887-019-1534-3

**Published:** 2019-05-24

**Authors:** Bruna Gonçalves C. da Silva, Mariângela Freitas da Silveira, Paula Duarte de Oliveira, Marlos Rodrigues Domingues, Nelson Arns Neumann, Fernando C. Barros, Andréa Dâmaso Bertoldi

**Affiliations:** 1 Postgraduate Program in Epidemiology, Federal University of Pelotas, Pelotas, RS Brazil; 2 Postgraduate Program in Physical Education, Federal University of Pelotas, Pelotas, RS Brazil; 3 National Coordination of the Pastoral da Criança, Curitiba, PR Brazil; 4 Postgraduate Course in Health and Behavior, Catholic University of Pelotas, Pelotas, RS Brazil

**Keywords:** Infant sleep position, Sudden infant death, Infant, Brazil, Cohort studies, Epidemiology, Public health

## Abstract

**Background:**

Non-supine infant sleep position is an important modifiable risk factor for sudden unexpected death in infancy. The aim of this study was to assess the prevalence of supine sleep position and associated factors among 3-month-old infants from a birth cohort in the city of Pelotas, southern Brazil.

**Methods:**

The present study evaluated longitudinal data from the 2015 Pelotas Birth Cohort. Study outcome was supine infant sleep position, defined as the appropriate position, among 3-month-old children. Demographic, socioeconomic, behavioral, and health characteristics collected at birth and at the 3-month follow-up were investigated as possible associated factors. The prevalence of each associated factor was investigated, and crude and hierarchical adjusted analyses were performed using Poisson regression.

**Results:**

Among the 4108 infants assessed in this study, 2274 (55.4%) slept in supine position at 3 months and only 66 (1.6%) in prone position. Maternal white skin color, higher family income and maternal schooling, advanced maternal age, maternal cohabiting with a partner, receiving counseling from health care professionals and non-bed-sharing were associated with higher prevalence of infants sleeping in supine position at 3 months. All these variables remained associated in our hierarchical adjusted analyses except maternal cohabitation with a partner. Participants with white mothers were more likely to sleep in supine position (PR: 1.23; 95%CI: 0.75–0.89) compared to participants with black mothers. Those belonging to the richest quintile were more likely to sleep in supine position (PR: 1.49; 95%CI: 1.35–1.65) compared to those who belong to the poorest. Mothers aged 31–36 years were more likely to choose supine sleep position (PR: 1.65; 95%CI: 1.42–1.92) compared to mothers younger than 19 years.

**Conclusions:**

The findings of the present study showed the influence of maternal age, socioeconomic status, and counseling on infant sleep habits as predictors of choice of infant sleep position in a Brazilian population. It is recommended to implement informative campaigns and public policies to at-risk population and to improve recommendations from health care professionals.

## Background

Infant sleep position is associated with sudden unexpected death in infancy (SUDI), which includes sudden infant death syndrome (SIDS) and other sleep-related deaths [1–4]. In the early 1990s, non-supine infant sleep position was identified as an important modifiable risk factor for SUDI [5]. After the success of international public health campaigns advising on the safe infant sleep position, there was a decrease in mortality rates from SUDI in many countries [6–8].

In 2009, a Brazilian non-governmental organization (*Pastoral da Criança*) officially launched a national awareness campaign about the safest sleep position for infants. A study with retrospective data collected in the city of Pelotas (Brazil) from 2006 to 2013 found a mortality coefficient by SIDS of 1.5 per thousand live births in this period (37 cases). Among these deaths, only 19% of the mothers reported adoption of supine sleep position [9]. The most recent data in Brazil showed a mortality coefficient by SUDI of 0.4 per thousand live births in 2016 (1093 cases) [10], considering the six International Classification of Diseases-10 (ICD-10) codes proposed by Taylor et al. [11]. However, vital statistics in Brazil are not accurate and have shown a high number of ill-defined causes of death [12], which means that the number of deaths by SUDI may be higher than the official statistics report.

Studies on the topic conducted in different countries have shown association between adopting the recommended infant sleep position and socioeconomic factors, especially maternal education level. However, the results for this association are different across countries. Studies carried out in the United States and in Canada found that higher educated mothers are more likely to place infants to sleep in the supine position [8, 13–15], whereas a study in Thailand found that higher educated mothers were more likely to place them in the prone sleep position [16].

It is very important for the development of public health policies that researchers evaluate the prevalence of use of the appropriate infant sleep position and its possible associated demographic, socioeconomic, and behavioral factors in Brazil years after the implementation of international and national campaigns. Therefore, the aim of the present study was to evaluate the prevalence of supine sleep position and its associated factors among 3-month-old infants from a cohort in the city of Pelotas, southern Brazil.

## Methods

All hospital-delivered live-born infants in Pelotas from January 1st, 2015 to December 31, 2015 were eligible for inclusion in the 2015 Pelotas birth cohort as long as their mothers lived in the urban area of the city, in Colônia Z3, and in Jardim América neighborhood (located in the neighboring city of Capão do Leão). The recruitment of participants and the follow-up started during the prenatal period, when a team of trained interviewers visited locations where pregnant women would potentially seek assistance during pregnancy (i.e. hospitals, ultrasound clinics, laboratories, university clinics and private obstetric and/or gynecological clinics) to identify pregnant women expecting to give birth in Pelotas in 2015. During that year, all mothers of live-born infants were invited to join the cohort. Immediately after delivery, those who agreed to participate were interviewed, and their children underwent anthropometric assessment. Afterwards, follow-up home visits were carried out three, twelve, and twenty-four months after delivery. During these visits, both mothers and children had anthropometric measures taken and parents or caregivers answered a questionnaire on demographic, socioeconomic, behavioral, and health aspects. Details on the methods and logistics of the 2015 Pelotas birth cohort can be found elsewhere [17]. At the 3-month follow-up, mothers or legal guardians were asked the following question: “In what position does the child usually sleep?”. For the present study, answers were dichotomized into appropriate and inappropriate infant sleep position, with appropriate position defined as supine sleep position.

Demographic, socioeconomic, behavioral, and health variables collected at birth and at the 3-month follow-up were investigated as factors possibly associated with supine infant sleep position at 3 months of age. The possible associated factors collected at birth were: infant sex; self-reported skin color (black, brown, white); maternal age (≤ 18, 19–24, 25–30, 31–36, ≥ 37 years); monthly family income (quintiles); maternal education (0–4, 5–8, 9–11, ≥ 12 years of complete schooling); parity (1, 2, 3, 4, 5 or more children); and maternal cohabitation with a partner. Other factors collected at the 3-month follow-up were: who advised on infant sleep position (nobody, child’s father or grandparents, physician, other health care professional, other), and bed sharing at 3 months of age (child sleeps in the same bed with another person - yes/no).

The characteristics of the sample were expressed as absolute (n) and relative (%) frequency. The prevalence of supine sleep position was expressed as percentage of the sample and its respective 95% confidence intervals (95%CI). Crude and adjusted hierarchical analyses to investigate the association between supine sleep position and the remaining variables were conducted using Poisson regression [18]. The adjusted model was developed by including variables with *p* < 0.20 in hierarchical levels. To avoid collinearity between family income and maternal education in the adjusted analysis, only family income was included in the hierarchical model (based on the best fit of the final model). Maternal skin color was entered into the first level of the hierarchical model; family income into the second; maternal age, parity and maternal cohabitation with a partner into the third; counseling on infant sleeping position into the fourth. Finally, bed sharing at 3 months of age was inserted into the last level. In this hierarchical model of analysis, variables were adjusted for those of superior hierarchical levels and of the same level. In addition to these analyses, the association between the type of health care coverage for childbirth (Brazilian Unified Health System - *Sistema Único de Saúde* [SUS], private health insurance or self-pay) and counseling on infant sleep position from a health care professional was assessed using the chi-square test. All analyses were performed using the statistical package Stata 13 (Stata Corporation, College Station, TX, USA). The level of significance was set at 5%.

The 2015 Pelotas birth cohort study was approved by the Research Ethics Committee of the School of Physical Education of Federal University of Pelotas (registration no. 26746414.5.0000.5313). The parents or legal guardians of all infants included in the cohort provided written informed consent for participation.

## Results

Of 4333 eligible births occurring in 2015, 4275 (98.7%) infants participated in this study. After 3 months, 4110 participants were successfully contacted for follow-up, representing a follow-up rate of 97.2% (considering 46 deaths). Overall, 4108 (94.8%) participants provided complete information on the study outcome and comprised the present sample. Table 1 describes the main characteristics of mothers and children included in the sample. Among the children investigated, 50.5% were male. With regard to maternal characteristics, most mothers reported white skin color (71.2%), had from 9 to 11 years of schooling (34.4%), were 25–30 years old (29.2%), had only one child (49.5%), and lived with a partner (85.8%). Most mothers were breastfeeding their child (76.7%), did not receive counseling on infant sleep position (41.6%) and did not report bed sharing (53.2%). The mean prevalence of children who usually slept in supine position was 55.4% (95%CI: 53.8–56.9).

**Table 1:** Description of study sample according to characteristics collected at perinatal and 3-month follow-up

Characteristics	n (%)
Infant’s sex	
Male	2076 (50.5)
Female	2032 (49.5)
Maternal skin color	
Black	646 (15.9)
Brown	527 (12.9)
White	2902 (71.2)
Family income (quintiles)	
Q1	762 (19.7)
Q2	778 (20.1)
Q3	778 (20.1)
Q4	869 (22.5)
Q5	680 (17.6)
Maternal education (years of schooling)	
0–4	367 (8.9)
5–8	1058 (25.8)
9–11	1413 (34.4)
≥12	268 (30.9)
Maternal age (years)	
≤18	417 (10.1)
19–24	1156 (28.1)
25–30	1198 (29.2)
31–36	988 (24.1)
≥37	348 (8.5)
Parity	
≥5	159 (3.9)
4	186 (4.5)
3	456 (11.1)
2	1273 (31.0)
1	2033 (49.5)
Maternal cohabitation with a partner	
No	584 (14.2)
Yes	3523 (85.8)
Who advised on infant sleep position*	
Nobody	1706 (41.6)
Child’s father or grandparents	157 (3.8)
Physician	1452 (35.4)
Other health care professionals	671 (16.3)
Other	120 (2.9)
Breastfeeding at 3 months*	
No	957 (23.3)
Yes	3150 (76.7)
Bed sharing at 3 months*	
Yes	1845 (46.8)
No	2096 (53.2)
Usual infant sleep position*	
Prone	66 (1.6)
Lateral	1768 (43.0)
Supine (appropriate position)	2274 (55.4)

*Variables collected at 3-month follow-up

*n* = 4108

Higher prevalence of supine infant sleep position was associated with maternal white skin color, higher family income, higher maternal schooling, maternal age from 31 to 36 years old, maternal cohabitation with a partner, and non-bed-sharing (Table 2). The highest prevalence of supine sleep position was observed among participants belonging to the richest quintile of family income (67.5%), and the lowest prevalence was observed among mothers younger than age 19 (36.2%). As for counseling on infant sleep position, the highest prevalence of supine sleep position was observed among mothers who received guidance from health care professionals other than physicians (65.1%), physicians (66.3%) and from people other than health care professionals and child’s father or grandparents (74.2%). In crude analyses, infant sex, parity and breastfeeding at 3 months were not significantly associated with supine sleep position (Table 2).

**Table 2:** Prevalence and crude prevalence ratios of supine sleep position among 3-month-old infants

Characteristics	Prevalence % (95%CI)	PR (95%CI)
Infant’s sex		*p = 0.341*
Male	54.6 (52.5–56.8)	1.00 (Ref.)
Female	56.1 (53.9–58.3)	1.03 (0.97–1.09)
Maternal skin color		*p < 0.001*
Black	47.7 (43.8–51.5)	1.00 (Ref.)
Brown	48.6 (44.3–52.8)	1.02 (0.90–1.15)
White	58.4 (56.6–60.2)	1.23 (1.12–1.34)
Family income (quintiles)		*p < 0.001*
Q1	43.4 (39.9–47.0)	1.00 (Ref.)
Q2	53.2 (49.7–56.7)	1.23 (1.10–1.36)
Q3	57.8 (54.4–61.3)	1.33 (1.20–1.47)
Q4	56.5 (53.2–59.8)	1.30 (1.18–1.44)
Q5	67.5 (64.0–71.0)	1.55 (1.41–1.71)
Maternal education (years of schooling)		*p < 0.001*
0–4	42.5 (37.4–47.6)	1.00 (Ref.)
5–8	44.9 (41.9–47.9)	1.06 (0.92–1.21)
9–11	56.1 (53.5–58.6)	1.32 (1.16–1.50)
≥12	67.0 (64.4–69.5)	1.58 (1.39–1.79)
Maternal age		*p < 0.001*
≤18	36.2 (31.5–40.8)	1.00 (Ref.)
19–24	48.5 (45.6–51.4)	1.34 (1.16–1.54)
25–30	57.4 (54.6;60.2)	1.59 (1.38–1.82)
31–36	65.9 (62.9–68.8)	1.82 (1.59–2.08)
≥37	63.8 (58.7–68.9)	1.76 (1.52–2.05)
Parity		*p = 0.172*
≥5	51.6 (43.8–59.4)	1.00 (Ref.)
4	53.8 (46.6–61.0)	1.04 (0.83–1.27)
3	59.4 (54.9–63.9)	1.15 (0.97–1.36)
2	56.6 (53.8–59.3)	1.09 (0.93–1.28)
1	54.1 (51.9–56.3)	1.05 (0.90–1.23)
Maternal cohabitation with a partner		*p < 0.001*
No	44.9 (40.8–48.9)	1.00 (Ref.)
Yes	57.1 (55.4–58.7)	1.27 (1.16–1.40)
Who advised on infant sleep position		*p < 0.001*
Nobody	43.1 (40.7–45.4)	1.00 (Ref.)
Child’s father or grandparents	31.8 (24.5–39.2)	0.74 (0.58–0.94)
Physician	66.3 (63.8–68.7)	1.54 (1.44–1.64)
Other healthcare professional	65.1 (61.5–68.7)	1.51 (1.40–1.63)
Other	74.2 (66.3–82.0)	1.72 (1.53–1.94)
Breastfeeding at 3 months*		*p = 0.132*
No	53.2 (50.0–56.4)	1.00 (Ref.)
Yes	56.0 (54.3–57.8)	1.05 (0.98–1.13)
Bed sharing at 3 months of age		*p < 0.001*
Yes	46.1 (43.8–48.3)	1.00 (Ref.)
No	62.1 (60.0–64.1)	1.35 (1.27–1.43)

95%CI: 95% confidence interval; PR prevalence ratio

n = 4108

Table 3 describes results for adjusted hierarchical analysis. The only two variables that did not remain in the hierarchical model were parity and breastfeeding (*p* > 0.20). The variables that were significantly associated with supine infant sleep position in crude analysis remained associated in adjusted analysis, except for maternal cohabitation with a partner. Mothers with white skin color were 23% (PR: 1.23; 95%CI: 1.12–1.34) more likely to choose supine position for their child to sleep compared to black mothers. There was no difference between maternal brown and black skin color. Children from families belonging to the richest quintile were 49% (PR: 1.49; 95%CI: 1.35–1.65) more likely to sleep in supine position compared to those in the poorest quintile. Conversely, mothers older than 18 were from 29 to 65% more likely to adopt supine sleep position compared to younger mothers. Mothers who received counseling on sleep position from health care professionals or people other than child’s father or grandparents were from 43 to 62% more likely to place their child in supine sleep position compared to mothers who did not receive counseling. Furthermore, children who do not share the same bed with someone else at 3 months of age were 20% (PR: 1.20; 95%CI: 1.12–1.27) more likely to sleep in supine sleep position compared to children who share their bed.

**Table 3:** Adjusted prevalence and prevalence ratios of supine sleep position among 3-month-old infants using hierarchical model of analysis

Hierarchical levels and variables	Prevalence % (95%CI)*	PR (95%CI)*
*Level 1*		
Maternal skin color		*p < 0.001*
Black	47.7 (43.8–51.5)	1.00 (Ref.)
Brown	48.6 (44.3–52.8)	1.02 (0.90–1.15)
White	58.4 (56.6–60.2)	1.23 (1.12–1.34)
*Level 2*		
Family income (quintiles)		*p < 0.001*
Q1	44.2 (40.6–47.9)	1.00 (Ref.)
Q2	53.7 (50.1–57.2)	1.21 (1.09–1.35)
Q3	58.2 (54.7–61.7)	1.32 (1.19–1.46)
Q4	56.1 (52.8–59.4)	1.27 (1.15–1.40)
Q5	66.1 (62.5–69.6)	1.49 (1.35–1.65)
*Level 3*		
Maternal age		*p < 0.001*
≤18	38.5 (33.2–43.9)	1.00 (Ref.)
19–24	50.0 (46.9–53.1)	1.29 (1.11–1.51)
25–30	56.7 (53.8–59.6)	1.47 (1.27–1.71)
31–36	63.7 (60.7–66.8)	1.65 (1.42–1.92)
≥37	61.9 (56.8–67.1)	1.61 (1.36–1.89)
Maternal cohabitation with a partner		*p = 0.060*
No	51.0 (46.1–55.8)	1.00 (Ref.)
Yes	56.2 (54.5–57.8)	1.10 (1.00–1.22)
*Level 4*		
Who advised on infant sleep position		*p < 0.001*
Nobody	44.5 (42.0–46.9)	1.00 (Ref.)
Child’s father or grandparents	34.6 (26.4–42.9)	0.77 (0.61–0.99)
Physician	63.5 (61.0–65.9)	1.43 (1.33–1.54)
Other health care professional	66.2 (62.5–70.0)	1.49 (1.38–1.61)
Other	72.0 (64.7–79.3)	1.62 (1.44–1.81)
*Level 5*		
Bed sharing at 3 months of age		*p < 0.001*
Yes	49.2 (46.8–51.7)	1.00 (Ref.)
No	59.0 (56.9–61.1)	1.20 (1.12–1.27)

95%CI: 95% confidence interval; PR prevalence ratio

*Adjusted for all variables of superior levels and of the same level

**Fig. 1:**
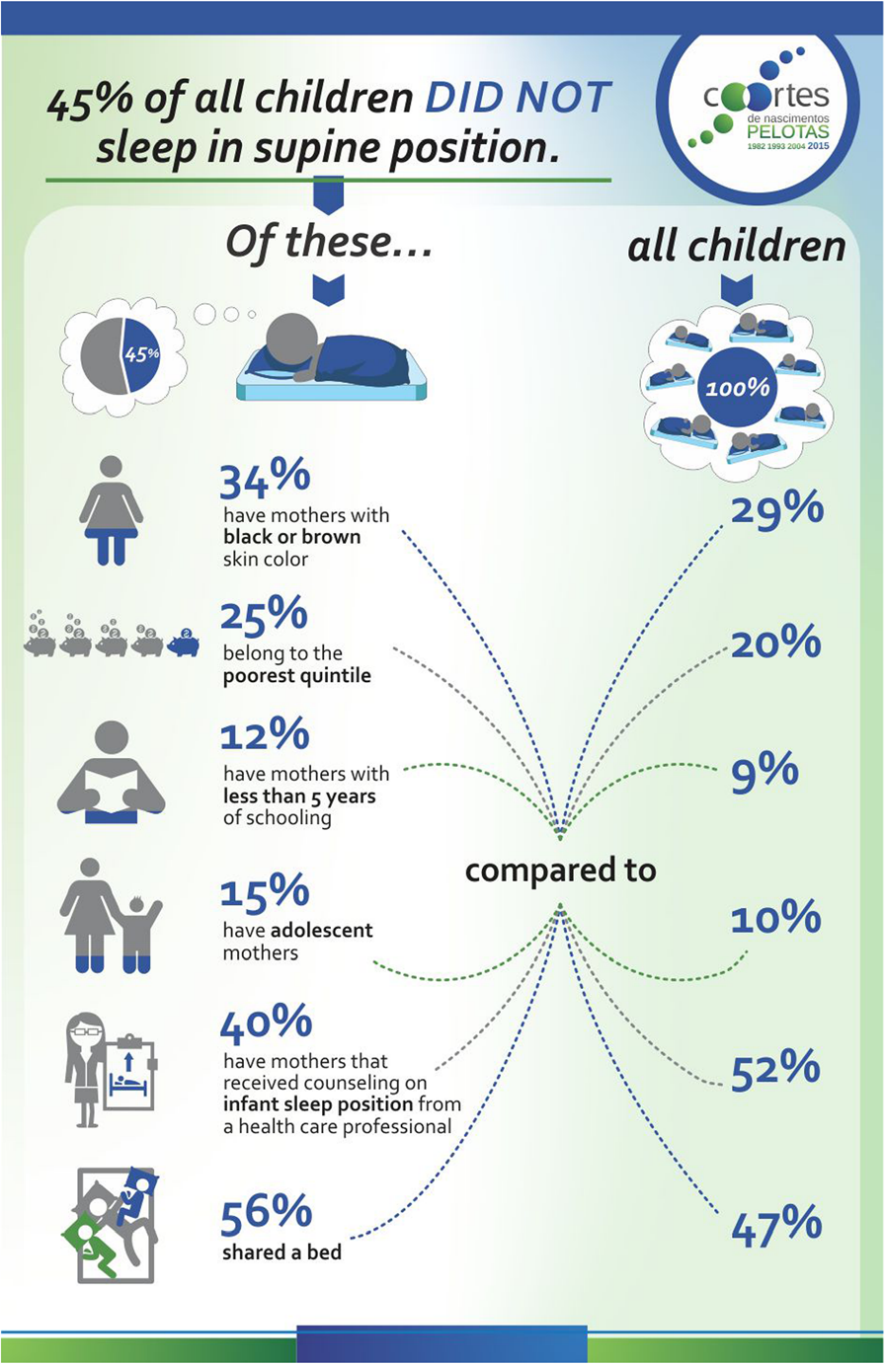
Infographic describing characteristics of who are sleeping in inappropriate position. 2015 Pelotas (Brazil) Birth Cohort

Figure 1 shows an infographic that describes characteristics of those that are sleeping in nonsupine sleep position. Higher prevalence of maternal brown or black skin color, families belonging to the poorest quintile, mothers with less than 5 years of education, adolescent mothers and bed-sharing were found in children sleeping in nonsupine sleep position compared to all children in the sample. Also, only 40% of mothers of those children have received counseling on infant sleep position from a health care professional compared to 52% of all children.

The percentage of mothers who received counseling on infant sleep position varied significantly according to the type of health care coverage for childbirth (Table 4). Mothers who had their delivery covered by the Brazilian Unified Health System were those who received less counseling from health care professionals (48.2%), whereas mothers covered by private health insurance were those who received more counseling from these professionals (61.7%).

**Table 4:** Heath care coverage for childbirth and counseling on infant sleep position from a health care professional

Type of health care coverage for childbirth	Counseling from a health care professional	p
	Prevalence % (95%CI)	0.001
SUS	48.2 (46.3–50.0)	–
Out-of-pocket	54.5 (49.8–59.3)	–
Private health insurance	61.7 (58.4–64.9)	–

95%CI: 95% confidence interval; SUS: Brazilian Unified Health System (*Sistema Único de Saúde*)

*Chi-square test

## Discussion

The present study investigated the prevalence and associated factors of supine infant sleep position among 3-month-old infants. Data from a birth cohort in a city in southern Brazil showed that only 55.4% of mothers reported that their child slept in the supine position at 3 months of age. Higher prevalence of supine position was associated with higher maternal age and education, higher family income, and counseling from health care professionals. Conversely, lower prevalence was associated with black and brown maternal skin color and bed sharing.

Studies conducted in the last decade in other countries have found higher prevalence rates of supine infant sleep position compared with that of the present study. Studies in the United States reported prevalence rates ranging from 63 to 72% [8, 19, 20], whereas a population-based study in Canada found a prevalence of 77% [15] and a study in Thailand observed that 60% of parents place their child to sleep in the supine position [16].

Differences in prevalence rates may be explained by socioeconomic and cultural differences in the populations assessed in these studies. For example, among the studies in United States, the study with the lowest prevalence (63%) investigated an exclusively African-American population, which has a less favorable socioeconomic status than the general American population [20]. In the study conducted in Thailand, Buddhist mothers, who accounted for nearly 80% of the study sample, were more likely to place their child to sleep in inappropriate positions [16]. There are no recent Brazilian studies assessing the prevalence of appropriate infant sleep position that could be used for comparison with the present study. However, a study conducted in another city in southern Brazil (Rio Grande) that investigated mothers’ intention to place infant in supine sleep position found that, in 2010, only 20% of mothers had this intention [21]. Another study conducted in the city of Rio Grande, but investigating maternal knowledge of infant sleep position found that, in 2013, 80.5% of mothers believed the best infant sleep position was sidelying [22].

Skin color and socioeconomic status are strong risk factors for SUDI [6, 23]. Several studies that investigated factors associated with infant sleep position found that skin color [8, 13, 14, 24, 25], income [14], education level [8, 13–15], and maternal age [26] were associated with infant sleep position. These findings corroborate the results obtained in the present study, which observed that maternal black or brown skin color, lower family income, lower education level, and younger maternal age were associated with lower prevalence of infants sleeping in supine position. Poor women are less prone to receive or give priority to information provided by health care professionals, since they usually spend little time with these professionals and their resources are scarce or spent in other items they deem more important to their family [14]. In addition, women with lower schooling may be unaware of the importance of placing their child to sleep in supine position or have difficulty in accessing and understanding the available educational material [14]. These factors may explain the disproportionately high SUDI burden among individuals with a disadvantaged socioeconomic status [15].

In addition to sleep position, bed sharing is also strongly associated with increased risk for SUDI, in some circumstances [27–29]. Previous studies showed that bed sharing is associated with unfavorable socioeconomic status [30, 31]. In our study, bed sharing at 3 months of age remained associated with lower prevalence of supine sleep position after adjusting for socioeconomic factors. These findings reinforce the importance of disseminating information on behaviors related to child’s sleep in the first year of life.

Several studies found a direct association between receiving counseling on infant sleep position from health care professionals and choice of supine sleep position [20, 30, 32, 33]. In the present study, counseling on infant sleep position from health care professional was associated with higher probability of using supine position, whereas counseling from child’s father or grandparents was associated with lower probability. It is important to highlight that this study did not investigate the type of counseling provided. Thus, health professionals may have recommended on the appropriate infant sleep position, based on their knowledge of the association between sleep position and SUDI, whereas child’s father and grandparents may have recommended inappropriate positions, based on cultural aspects and beliefs. Moreover, there was a difference in the prevalence rates of counseling on infant sleep position from health care professional according to the type of health care coverage for childbirth, with mothers covered by the SUS receiving little advice. This finding highlights inequalities in antenatal and perinatal care.

Although counseling from health care professionals is associated with changes in parents’ behavior [8, 19, 33], some studies showed that beliefs on the possibility of increasing the incidence of aspiration or asphyxia in supine position and concern on infant’s comfort are other determinant factors in parents’ choice of infant sleep position, although being aware of the recommended infant sleep position [8, 19, 33, 34].

An important finding of this study was also the low prevalence of usual prone sleep position. Only 1.6% of the mothers reported that the child usually sleeps in prone position. On the other hand, 43% reported usual lateral sleep position. The high prevalence of lateral sleep position could be explained by the fact that infants may be placed in the lateral position to further facilitate breastfeeding and bed sharing would be also a way to facilitate this practice [29]. However, breastfeeding was not associated with sleep position in our crude and adjusted analysis. Based on our data, since the prevalence of prone position was very low, we can deduce that usual lateral sleep position was associated with black and brown mothers, infants belonging to poorest families, young mothers, mothers receiving advice on infant sleep position by the child’s father or grandparents and bed sharing.

National campaigns are extremely important to raise awareness on the topic. A campaign launched in the United States in 1994, known as “Back to Sleep”, was associated with reduced SUDI incidence. Studies that investigated temporal trends in sleep position in the United States showed that, after this campaign, there was an increase in the number of children who were placed in supine sleep position [8, 25]. In Brazil, a national campaign launched by the *Pastoral da Criança* in 2009 promoted the supine infant sleep position. Studies conducted before this national campaign found much lower prevalence rates of children placed to sleep in supine position. A study conducted in 2004 in the city of Passo Fundo showed that, in that year, only 4.3% of children slept in supine position [35]. Additionally, data from 2004 Pelotas (Brazil) Birth Cohort revealed that, in that year, only 20.8% of mothers reported to place their child to sleep in supine position and 4.7% in prone position at 3 months of age (unpublished data), while 6 years after the national campaign 55.4% of mothers from the 2015 Pelotas (Brazil) Birth Cohort reported supine sleep position and 1.6% use of the prone sleep position.

The present study has several strengths. The data come from a population-based study with a high follow-up rate, thus minimizing the risk of selection bias. The findings may be generalized to southern Brazil population. Additionally, data were collected prospectively, which enabled us to establish a temporal relation between exposures and outcome. The large number of collected variables also allowed for a detailed hierarchical analysis of associated factors.

However, some limitations should be mentioned. First, this study investigated whether mother received counseling on infant sleep position and who provided it, but we did not have information on which position was recommended. Thus, it is not possible to state that mothers who received counseling were actually aware of the appropriate sleep position. Second, mothers were not asked about the reason for choosing a given sleep position; hence, it was not possible to identity the major determining factor for this choice. Although breastfeeding status has not been associated with supine sleep position in our sample, it would be important to know if the lateral sleep position was chosen to facilitate breastfeeding, for example, since in this case such behavior should not be discouraged [29]. Finally, mothers were asked about usual infant sleep position rather than about an exclusive or particular behavior (i.e. in what position the mother put the child to sleep last night), which may have contributed to a possible underestimation of the investigated associated factors and also of the nonsupine sleep position prevalence [36].

## Conclusions

The results of the present study showed that many people still do not follow the guidelines regarding infant sleep position in the first months of life. Maternal brown or black skin color, younger maternal age, lower family income, lower maternal schooling, absence of counseling from health care professionals, and bed sharing were associated with lower probability of infants sleeping in the supine position. These findings show the need to improve appropriate counseling from health care professionals and importance of implementing new campaigns and public policies on infant sleep position, especially those aimed to women at risk of choosing inappropriate infant sleep positions, in order to decrease the incidence of SUDI.

## Abbreviations

CI: confidence interval
PR: prevalence ratio
SUDI: sudden unexpected death in infancy
SUS: Brazilian Unified Health System - *Sistema Único de Saúde*
